# ACE/ACE2 axis in cardiovascular disease and COVID-19: Molecular insights and therapeutic perspectives

**DOI:** 10.34172/jcvtr.026.33335

**Published:** 2026-03-30

**Authors:** Sony Peter, Thiruchenduran Mohana, Dhanavel Anandhi, Vishwanathan Sathya Priya, Romi Keerikkattil Sleeba, Harisree Panikkaveedu Haridas, Midhun Thazhissery Mohanan, Swathi Thoduvayil, Sinha Mathew, Dhanya Muraleedharan Santhamma, Dinesh Roy Divakaran

**Affiliations:** ^1^Meenakshi Academy of Higher Education and Research (Deemed to be University), Chennai, Tamil Nadu, India; ^2^Department of Biochemistry, Meenakshi Ammal Dental College & Hospital, Meenakshi Academy of Higher Education and Research (Deemed to be University), Chennai, Tamil Nadu, India; ^3^Meenakshi Ammal Dental College & Hospital, Meenakshi Academy of Higher Education and Research (Deemed to be University), Chennai, Tamil Nadu, India; ^4^Department of Biochemistry, ACS Medical College & Hospital, Chennai, Tamil Nadu, India; ^5^Centre for Advanced Genetic Studies, Thiruvananthapuram, Kerala, India

**Keywords:** Angiotensin-converting enzyme, Angiotensin-(1-7), COVID-19, Heart failure, Myocardial tissue, Renin-angiotensin system

## Abstract

The renin-angiotensin system (RAS) plays a central role in regulating blood pressure and cardiovascular health. Angiotensin-converting enzyme (ACE) facilitates the conversion of angiotensin I to angiotensin II, a potent vasoconstrictor that contributes to hypertension and heart failure. Conversely, ACE2 converts angiotensin II into angiotensin-(1-7), a vasodilator with protective cardiovascular effects. An imbalance between ACE and ACE2 activities has been increasingly associated with the progression of cardiovascular diseases and complications related to COVID-19. This review analyzed 100 relevant studies published up to May 2024, identified through a comprehensive literature search on PubMed and Scopus. The findings highlighted that dysregulation of the ACE/ACE2 axis exacerbates cardiovascular dysfunction. The interaction of SARS-CoV-2 with ACE2 reduces its protective function, intensifying inflammatory responses and leading to complications such as lung injury and heart failure. Additionally, genetic polymorphisms in ACE and ACE2 influence individual susceptibility and severity of COVID-19. Promising therapeutic strategies, including ACE2-based peptides and angiotensin II receptor modulators, are under investigation but require further clinical validation. Targeting the ACE/ACE2 axis could provide effective treatment options for cardiovascular disease and COVID-19-related complications, warranting further in-depth research.

## Introduction

 The renin-angiotensin system (RAS) regulates fluid and salt balance, with ACE converting angiotensin I to angiotensin II, which causes vasoconstriction and controls blood pressure. ACE inhibitors are essential in treating cardiovascular diseases like hypertension and heart failure. ACE2, which balances ACE, contributes to cardiovascular health and impacts diseases like COVID-19. Dysregulation of the ACE/ACE2 balance not only facilitates viral entry in COVID-19, but also exacerbates cardiovascular dysfunction, inflammation, and tissue remodelling in conditions such as hypertension, atherosclerosis, myocardial infarction, and heart failure. A shift toward elevated ACE activity with reduced ACE2 expression intensifies vasoconstriction, oxidative stress, and fibrosis, thereby aggravating disease progression across both infectious and non-infectious cardiovascular pathologies. Understanding ACE expression and regulation is crucial for insights into health and disease.^1^ In heart failure, RAS dysregulation and altered ACE expression contribute to disease progression. ACE, discovered by Skeggs et al is highly expressed in the heart and other tissues.^2^ Studies by Metzger et al and others have shown ACE’s involvement in local RAS activity.^3^ ACE’s interaction with zinc, governed by the active site HEXXH, underscores its importance in cardiac physiology. Altered ACE expression in heart failure can lead to pathological remodelling. Increased ACE activity is linked to kidney and cardiovascular disease progression, as noted by Trojanowicz et al^4^ ACE in leukocytes suggests a role in modulating immune responses via RAS components, affecting disease progression. ACE1 polymorphisms are associated with systolic heart failure, indicating genetic susceptibility.^5^ The balance between ACE and ACE2, which converts angiotensin II to vasodilatory angiotensin (1–7), is crucial in heart failure. Studies highlight ACE2’s role in myocardial remodelling and heart failure, suggesting a potential therapeutic target. Understanding ACE regulation in heart failure provides insights into disease mechanisms and possible treatments.^6,7^

 Despite extensive research, existing studies often examine ACE or ACE2 in isolation, lacking an integrated molecular view of their roles in both cardiovascular disease and COVID-19. Given ACE2’s dual role in cardiovascular protection and viral entry, a comprehensive assessment of its gene variants, expression, and function is critical. This review aims to consolidate current evidence on ACE/ACE2 dysregulation, highlighting their molecular mechanisms, disease associations, and therapeutic implications.

## Materials and Methods

###  Primary research question

 What is the association between angiotensin-converting enzymes (ACE and ACE2) and their genetic polymorphisms with cardiovascular diseases, COVID-19 severity, aging, and inflammatory conditions in humans?

###  Time frame

 This review includes studies published from covering studies up to May 2024 to capture relevant advances in the genetics and clinical aspects of ACE and ACE2.

###  PICOS Framework

####  Population (P)

 Human subjects of any age or gender, with or without cardiovascular diseases, COVID-19, aging-related or inflammatory disorders.

####  Intervention/ Exposure

 Presence of ACE or ACE2 gene polymorphisms, gene expression, or protein level variations.

####  Comparison

 Comparison between individuals with and without ACE/ACE2 polymorphisms or altered expression levels, or healthy controls vs affected patients.

####  Outcomes

 Clinical outcomes (disease incidence, severity, and mortality), biochemical parameters, gene expression levels, or molecular biomarkers related to ACE/ACE2.

####  Study Design

 Observational studies (cohort, case-control, cross-sectional), randomized controlled trials, and experimental studies involving human subjects.

###  Search strategy

 A comprehensive literature search was conducted using multiple electronic databases, including PubMed/MEDLINE, Embase, Scopus, Web of Science, and the Cochrane Library, to identify relevant studies.

###  Selection criteria

####  Inclusion

 The inclusion criteria for this review comprised studies conducted on human subjects that evaluated ACE or ACE2 gene polymorphisms, gene expression, or protein levels. Eligible studies reported associations between ACE/ACE2 and conditions such as cardiovascular diseases, COVID-19, aging-related disorders, or inflammatory conditions. The review included observational studies (cohort, case-control, cross-sectional), randomized controlled trials, and experimental designs involving human data. Only articles published in studies 1997 to 2024 were considered.

####  Exclusion

 The exclusion criteria included animal or in vitro studies that lacked human clinical data, as well as reviews, editorials, commentaries, case reports, and conference abstracts without full datasets. Studies were also excluded if they lacked a clear methodology, had incomplete information on ACE or ACE2, or were published in languages other than English.

###  Study selection

 Two independent reviewers screened titles and abstracts for eligibility. Full texts of potentially relevant articles were reviewed. Disagreements were resolved through discussion or by a third reviewer.

###  Data extraction

 Data were independently extracted by two reviewers using a standardized form that captured key details such as author, publication year, country, study design, population characteristics, specific ACE or ACE2 gene polymorphisms or expression data, measured outcomes, main findings, and study limitations. Any discrepancies between reviewers were resolved through discussion and consensus.

###  Risk of bias assessment

 Two reviewers independently performed the assessments, and any disagreements were resolved through consensus or consultation with a third reviewer.

###  Angiotensin-converting enzymes

 Angiotensin-converting enzyme (ACE) is essential in the renin-angiotensin system (RAS), cleaving angiotensinogen to produce angiotensin II.^1^ ACE is significantly expressed in various tissues.^6,8,9,10^ It is primarily located on cell membranes via a carboxy-terminal transmembrane domain. While genetic polymorphisms can influence serum ACE levels, stability is generally observed in adults, although Bénéteau-Burnat et al suggest higher levels in children.^11^

 Angiotensin-converting enzyme (ACE, EC 3.4.15.1) is a zinc-metallopeptidase on cell surfaces in various mammalian tissues. Its interaction with zinc is governed by the active site HEXXH. ^12,13^ This enzyme, as mentioned by Ehlers & Riordan catalyzes the removal of dipeptides from the C-terminus of short oligopeptides.^14^ ACE regulates blood pressure and electrolyte balance in mammals through the renin-angiotensin system. It has two forms: somatic ACE, with N- and C-domains, and testicular ACE, which has a single domain identical to the C-domain of somatic ACE and is found in spermatids and spermatozoa. Insects also possess ACE enzymes with enzymatic properties akin to mammalian ACEs, albeit differing structurally due to their soluble and minimally glycosylated proteins.^15-17^ Table 1 highlights how ACE2 mitigates the effects of ACE by transforming angiotensin II into a vasodilatory peptide, thereby contributing to cardiovascular protection.

**Table 1:** illustrate ACE2 counteracts the action of ACE by converting angiotensin II into angiotensin a vasodilatory peptide that provides cardiovascular protection.

**Enzyme**	**Function**	**Reference**
Angiotensin-converting enzyme (ACE)	ACE converts angiotensin I into the vasoconstrictor angiotensin II and breaks down bradykinin	^18^
Angiotensin-Converting Enzyme 2 (ACE2)	It opposes ACE by converting angiotensin II into angiotensin-(1-7), which promotes vasodilation and offers cardiovascular protection.	^19^
Endothelial Cell ACE	ACE in endothelial cells locally controls blood pressure and vascular function.	^8^

###  Functions of angiotensin-converting enzyme (ACE) 

 ACE contributes to blood pressure regulation by converting angiotensin I into angiotensin II. Studies in rodents and computer simulations, such as Smithies et al indicate that angiotensin II production is primarily regulated by renin. Despite requiring over 90% ACE inhibition to significantly reduce angiotensin II levels, pharmacological ACE inhibitors effectively lower blood pressure.^20^ Kim & Iwao found that extensive research has explored the impact of ACE and angiotensin II on blood pressure regulation, along with the diverse physiological functions of ACE due to the effects of angiotensin II and other ACE cleavage products.^21^

###  Gene variants of angiotensin-converting enzymes (ACE, ACE2) and COVID-19 severity 

 SARS-CoV-2, the virus behind COVID-19, belongs to the Sarbecovirus subgenus, as noted by Gorbalenya et al^22^ Similar to SARS-CoV, it is an ACE2-tropic virus, with the “spike” (S) protein binding to ACE2-expressing cells in the nasopharyngeal mucosa and alveolar pneumocytes.^23-25^ COVID-19 exhibits a spectrum of severity from mild to severe cases, as described by Fu et al and Rivieccio et al ^26,27^ Viral infections can cause severe inflammation, lung damage, and raise the risk of multi-organ failure and death. The RAAS plays a key role in COVID-19, with ACE promoting vasoconstriction, inflammation, and fibrosis, while ACE2 counteracts these effects by promoting vasodilation and reducing lung damage.^28-30^

 Hypertension and cardiovascular disease, prevalent in COVID-19 patients, raise hospitalization and mortality risk Zhou et al^31^ Both acquired and inherited factors affecting RAAS components can influence COVID-19 outcomes. ACE2 expression declines with age and is higher in men, potentially explaining increased risks in older and male individuals. Conditions reducing ACE2 expression may heighten hypertension and heart failure risk, while high ACE activity can elevate lung and cardiovascular disease risk by enhancing the Ang-II/AT1R axis.^32,33^ Marshall et al found that the ACE I/D polymorphism, particularly the D/D genotype, is linked to higher ACE levels and increased risk of hypertension and heart disease.^34^ The ACE2 gene, located on the X chromosome, may disadvantage male carriers of low-expression alleles, contributing to severe COVID-19 prevalence in males. SARS-CoV reduces myocardial ACE2 expression, explaining myocardial damage in SARS patients, according to Oudit et al^35^ Variants in ACE and ACE2 genes can influence COVID-19 symptoms and outcomes. Regional differences in allele frequencies may explain variations in COVID-19 incidence and mortality rates, as suggested by Yamamoto et al.^36^

**Figure 1:**
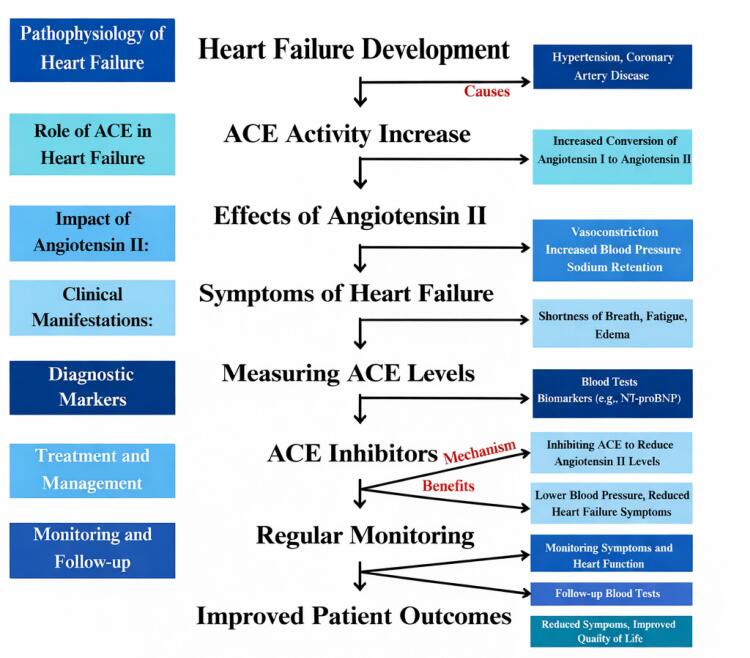
ACE Activity, Angiotensin II Effects, and Heart Failure Progression

Figure 1 depicts the sequential pathway of heart failure development, emphasizing the role of ACE and angiotensin II, associated symptoms, diagnostic markers, therapeutic intervention with ACE inhibitors, and the significance of continuous monitoring for better clinical outcomes.

###  Myocardial ACE2 protein expression in ischemic heart failure 

 The ACE2-angiotensin-Mas receptor axis is crucial in regulating myocardial remodeling and heart failure development, with ACE2 playing a central role.^33,37^ ACE2 converts angiotensin I to angiotensin-(1-7) and deactivates angiotensin II, both benefiting cardiovascular health.^38-43^ Angiotensin binds to MasR, influencing MAPK, PKB, and oxidative stress pathways.^44^ ACE2 reduces angiotensin II and AT1R levels, providing cardioprotection and promoting vasodilation during remodeling.^37^ ACE2 exists as both a membrane-bound protein and a soluble form, as noted by Xiao et al^45^ Increased plasma activity of soluble ACE2 correlates with adverse cardiac remodeling and serves as a prognostic marker for cardiovascular and all-cause mortality.^46-48^ Ectodomain shedding of ACE2 is vital in the cardiac remodeling process.^49,50^

###  Inflammatory role of ACE

 ACE1 and ACE2 are key regulators in the renin-angiotensin system (RAS).^51^ ACE1 increases ROS, causing inflammation and vasoconstriction, whereas ACE2 reduces inflammation and promotes vasodilation, impacting blood pressure and bodily functions.^52,53^ ACE2 converts angiotensin II to angiotensin-(1–7), which activates the Mas receptor pathway. This pathway is believed to exert protective effects, such as vasodilation and inhibition of fibrosis.^54^ SARS-CoV-2, the virus responsible for COVID-19, targets ACE2 receptors, disrupting the balance between ACE1 and ACE2 and leading to cardiovascular complications.^55^

 ACE1 is present in various tissues and also in soluble forms in urine, serum, seminal fluid, amniotic fluid, and cerebrospinal fluid.^52^ It is expressed in monocytes, macrophages, and T cells, with varying activity levels: low in monocytes, high in macrophages, and intermediate in T cells. In type 1 diabetes and obesity-related inflammation, ACE1 and ACE2 expression levels are altered: type 1 diabetes shows higher ACE1 and lower ACE2 levels, while obesity-related inflammation is associated with increased ACE1 mRNA and activity in T cells.^53^ These findings highlight ACE1’s role in immune modulation and inflammation, suggesting it as a potential therapeutic target for inflammatory conditions. Understanding the opposing roles of ACE1 and ACE2 provides insight into strategies for managing cardiovascular health and COVID-19.

###  Role of ACE1 in aging and age-related diseases 

 ACE1 expression’s link to aging, chronic inflammation, and age-related diseases has gained attention. Research indicates that ACE1 expression is associated with the progression of kidney and cardiovascular diseases, suggesting that circulating leukocytes play a role in modulating local immune responses through the renin-angiotensin system (RAS). Coppo et al found an inverse relationship between RAS gene expression in T cells and both serum inflammatory cytokine levels and serum insulin levels.^56^ Trojanowicz et al revealed that increased ACE1 expression in monocytes correlates with the progression of kidney and cardiovascular diseases, indicating a role for circulating leukocytes in modulating local immune responses through RAS components.^57^ Pawelec et al; Alves et al; and Bueno et al proposed ACE1’s role in aging-related conditions and chronic inflammation, such as Alzheimer’s, sarcopenia, and cancer.^58-60^ Kehoe et al and MacLachlan et al have associated ACE1 polymorphisms with an increased susceptibility to Alzheimer’s disease.^61,62^ Yoshihara et al reported increased ACE1 expression in brain homogenates during normal aging and its correlation with sarcopenia.^63^ Carl-McGrath et al and Zhang et al highlighted elevated ACE1 expression in cancer tissues compared to healthy tissues.^64,65^ Joshi et al showed that with aging, hematopoietic stem/progenitor cells display reduced ACE2 and heightened ACE1 expression, suggesting a shift toward the proinflammatory aspect of the local RAS.^66^ They also found varying ACE1 expression levels in different immune cell subsets, with higher expression observed in lymphoid cells.

**Figure 2:**
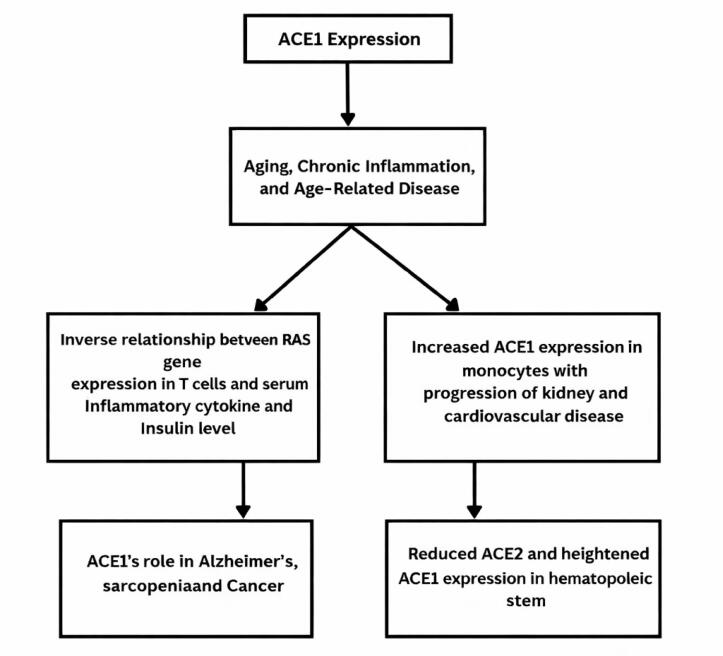
Influence of ACE1 Expression on Aging, Chronic Inflammation, and Age-Related Diseases

Figure 2 demonstrates the role of ACE1 expression in modulating aging and chronic inflammation, highlighting its effects on inflammatory cytokine production, insulin regulation, progression of kidney and cardiovascular diseases, and the maintenance of hematopoietic stem cell function.

###  Role of ACE2 in aging and age-related diseases 

 ACE2 plays a crucial role in aging and age-related diseases by regulating the renin-angiotensin system. Altered ACE2 expression in the elderly is linked to hypertension, cardiovascular diseases, neurodegenerative disorders like Alzheimer’s, metabolic syndrome, immune dysregulation, renal decline, and oxidative stress. These findings highlight ACE2 as a key factor in aging and a potential target for therapeutic interventions.

Table 2 presents an overview of the critical functions of ACE2 in aging and age-associated disorders, outlining its influence on multiple physiological pathways and health outcomes.

**Table 2:** summarizes the key aspects of ACE2’s role in aging and age-related diseases, highlighting its impact on various physiological processes.

**Aspect**	**Description**	**Reference**
Aging and Cardiovascular Health	Increased ACE2 activity is linked to hypertension and cardiovascular diseases in elderly populations.	^67,68^
Neurodegenerative Diseases	Elevated ACE2 levels are associated with Alzheimer's disease and other neurodegenerative conditions.	^69,70^
Metabolic Disorders	ACE2 contributes to insulin resistance and metabolic syndrome in aging individuals.	^71^
Immune System Modulation	Altered ACE2 expression affects immune response and inflammation in older adults.	^72^
Renal Function	ACE2 activity influences age-related decline in renal function and chronic kidney disease.	^73^
General Aging Process	ACE2 levels impact oxidative stress and cellular senescence, contributing to the overall aging process.	^74,75^

ACE/ACE2 balance in health vs. disease

## ACE/ACE2 balance in health vs. disease

 The renin-angiotensin system (RAS) plays a pivotal role in cardiovascular and renal physiology, where a delicate balance between ACE and ACE2 activities is essential for maintaining homeostasis. ACE converts angiotensin I to angiotensin II, a potent vasoconstrictor that promotes inflammation, fibrosis, and oxidative stress, contributing to the progression of hypertension, heart failure, and chronic kidney disease. In contrast, ACE2 acts as a counter-regulatory enzyme by degrading angiotensin II into angiotensin-(1-7), which exerts vasodilatory, anti-inflammatory, and anti-fibrotic effects, thereby protecting tissues from damage. Disruption of this balance, characterized by increased ACE activity and reduced ACE2 expression, is implicated in the pathogenesis of various age-related and chronic inflammatory diseases. Therapeutic approaches targeting the restoration of ACE/ACE2 equilibrium hold significant potential for mitigating cardiovascular and renal dysfunction. ^76^

**Figure 3:**
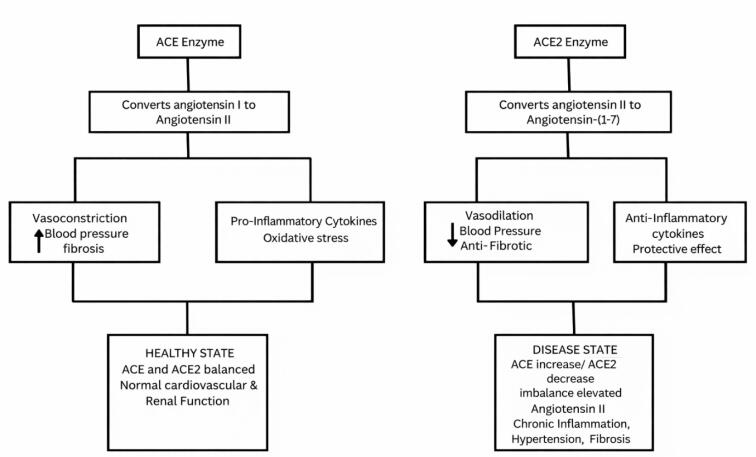
Influence of ACE1 Expression on Aging, Chronic Inflammation, and Age-Related Diseases

Figure 3 depicts the opposing actions of ACE and ACE2 enzymes in the renin-angiotensin system, emphasizing how their balance supports cardiovascular health while imbalance contributes to disease progression.

###  Role of ACE in heart failure

 ACE inhibitors play a crucial role in the management of heart failure by significantly reducing cardiovascular mortality, myocardial infarction (MI), and hospitalizations in patients with left ventricular (LV) systolic dysfunction. Evidence from the extended SOLVD (X-SOLVD) trial demonstrated a notable reduction in all-cause mortality with ACE inhibitor therapy (hazard ratio [HR] 0.86; *P* < 0.001), while the ATLAS study showed that high-dose lisinopril significantly reduced heart failure-related hospitalizations compared to low-dose therapy (*P* = 0.002). Despite their clinical benefits, ACE inhibitors do not fully suppress the renin-angiotensin-aldosterone system (RAAS), as angiotensin II can also be generated via non-ACE pathways. This has led to the evaluation of angiotensin receptor blockers (ARBs) such as candesartan and valsartan. While ARBs offer therapeutic alternatives, especially in ACE inhibitor-intolerant patients, ACE inhibitors remain the first-line treatment for patients with post-MI and systolic heart failure. ^77^

 At the cellular and molecular levels, heart failure resulting from conditions like MI, hypertension, or diabetes is marked by subcellular remodelling, metabolic dysfunction, and abnormalities in calcium handling within cardiomyocytes. Elevated levels of angiotensin II due to RAS activation contribute to oxidative stress and structural and functional deterioration of the heart. Studies have shown that treatment with ACE inhibitors such as enalapril or AT1 receptor antagonists like losartan can mitigate these alterations. These therapies improve cardiac function by attenuating subcellular remodelling, reducing oxidative stress, and suppressing RAS hyperactivity. Such evidence highlights the pivotal role of ACE and angiotensin II in driving the progression of heart failure through cellular and molecular pathways. ^78^

###  ACE activity and regulation in cardiovascular health

 The RAAS maintains fluid and salt balance, with ACE converting angiotensin I to active angiotensin II (AngII). Skeggs et al Williams & Zhang, 2020; Seferović et al 2019, first identified ACE, and ACE inhibitors are crucial in cardiovascular therapy, especially for hypertension and heart failure treatment.^1,79,80^ ACE2 counteracts the effects of AngII, playing a crucial role in cardiovascular health and COVID-19 pathology.^81,82^ Successful ACE inhibitors, noted for low lipophilicity, were discussed by.^83^ The I/D polymorphism of the ACE gene is linked to systolic heart failure, according to Chang et al^5^ Although primarily expressed in endothelial cells, ACE is also present in the human heart and other organs, affecting circulating ACE levels.^84,85^ Serum albumin acts as an endogenous inhibitor of circulating ACE, inhibiting its activity at physiological concentrations.^86^ This study explores how tissue ACE/ACE2 expression and circulating ACE/ACE2 activity in cardiovascular disease and COVID-19 are influenced by genetic expression, endogenous inhibition, and secretion mechanisms. Genetic polymorphisms and endogenous inhibitors are identified as regulators of circulating ACE activity, with specific impacts on cardiac function. Table 3 presents an overview of the function, mode of action, clinical effectiveness, and common side effects of ACE inhibitors in treating heart failure.

**Table 3:** outlines the therapeutic role, mechanism, clinical benefits, and potential adverse effects of ACE inhibitors in the management of heart failure

**Aspect**	**Description**	**Reference**
Role in Heart Failure	ACE inhibitors reduce heart failure mortality, hospitalizations, and symptoms by blocking angiotensin II formation from angiotensin I.	^87-89^
Mechanism of Action	ACE inhibitors inhibit the breakdown of bradykinin and decrease angiotensin II levels, leading to vasodilation, reduced aldosterone secretion, and decreased preload and afterload.	^90,91^
Clinical Efficacy	ACE inhibitors improve heart function and reduce symptoms of heart failure, such as dyspnea and fatigue.	^92^
Adverse Effects	Typical side effects include hypotension, renal impairment, hyperkalemia, and cough, attributed to elevated bradykinin levels.	^93^

###  Emerging therapeutics targeting the ACE/ACE2 axis

 The ACE/ACE2 axis has gained significant attention as a therapeutic target, especially in the context of the COVID-19 pandemic. Emerging therapies are being developed to modulate this axis, aiming to restore the balance between the pro-inflammatory effects of ACE1 and the protective, anti-inflammatory functions of ACE2.

####  ACE2-based therapies

 Peptides from ACE2’s N-terminal helix α1, designed to block SARS-CoV-2 binding, were shown to effectively bind to the virus spike proteins in simulations.^93^ Stapled ACE2 peptidomimetics were created to block the spike protein’s RBD, stopping it from binding to human ACE2 receptors.^94^ These polypeptides and peptidomimetic drugs are being explored as potential treatments to reduce COVID-19 hospitalization and mortality.^95^

####  ATII receptor modulators

 Researchers found ACE inhibitors (ACEIs) and angiotensin receptor blockers (ARBs) lower mortality and severe outcomes in COVID-19 patients.^96^ Additionally, stapled ACE2 peptidomimetics block SARS-CoV-2 spike protein binding to ACE2.^97^ Research is ongoing on ARBs’ benefits for COVID-19.

####  ACE inhibitors

 A study of 1.1 million patients found no increased COVID-19 risk with ACE inhibitors or ARBs, though ACE inhibitors had higher risks in Caribbean and Black African groups. ACE inhibitors and ARBs were both associated with a lower COVID-19 risk.^98^

####  Recent approvals and ongoing trials

 APN01 blocks SARS-CoV-2 entry and converts angiotensin II to angiotensin-(1-7), reducing inflammation and protecting lung and cardiovascular tissues. A Phase 2 trial assessed APN01 in severely ill COVID-19 patients. While the trial did not achieve statistical significance due to a low number of events, APN01 showed potential benefits, including an increase in mechanical ventilator-free days and a decrease in viral load.^99^ The BRACE CORONA trial examined if pausing ACE inhibitors or ARBs impacts COVID-19 outcomes, finding no difference in 28-day mortality between the losartan and usual care groups.^98^ Additional studies have examined how losartan affects lung injury in COVID-19 patients.^100^

 Despite the comprehensive review of literature on the role of angiotensin-converting enzymes (ACE and ACE2) in cardiovascular health, COVID-19, and aging-related diseases, several limitations must be acknowledged. First, the majority of the included studies are observational or experimental in vitro and animal models, which may limit direct clinical applicability in humans due to interspecies differences and controlled experimental conditions. Second, heterogeneity among studies in terms of population demographics, methodologies, and measurement techniques for ACE/ACE2 expression may affect the consistency and generalizability of the findings. Third, the rapidly evolving nature of COVID-19 research means that emerging data and novel variants of SARS-CoV-2 could influence the understanding of ACE2’s role in viral pathogenesis and cardiovascular complications beyond the scope of this review. Fourth, genetic polymorphisms of ACE and ACE2 genes show significant regional and ethnic variability, which may confound the interpretation of their association with disease susceptibility and outcomes in diverse populations. Additionally, the review primarily focuses on the molecular and genetic aspects of ACE/ACE2 without extensive analysis of potential environmental, lifestyle, and pharmacological interactions that could modify enzyme activity and disease progression. Finally, the exclusion of non-English language studies and case reports might have omitted valuable data and insights. Future research incorporating large-scale, longitudinal clinical studies with diverse populations and standardized methodologies is warranted to address these limitations and deepen understanding of ACE/ACE2 in health and disease.

## Conclusion

 The renin-angiotensin system (RAS) plays a key role in cardiovascular health, where ACE raises blood pressure and ACE2 counteracts this effect. Dysregulation of this balance, especially in heart failure, underscores their therapeutic relevance. Recent research emphasizes the influence of genetic variants in ACE and ACE2 on cardiovascular outcomes and COVID-19 severity. While ACE inhibitors effectively lower cardiovascular risk, their impact on COVID-19 remains complex, with ongoing trials assessing their safety and efficacy. This review provides a novel perspective by highlighting the underexplored dual roles of ACE and ACE2 not only in cardiovascular regulation but also in immune modulation, aging, and viral susceptibility. It underscores the emerging importance of the ACE1/ACE2 ratio as a potential biomarker and therapeutic target, while also drawing attention to genetic polymorphisms, tissue-specific expression, and soluble ACE2 as tools for future personalized interventions. Understanding this balance and its broader implications could enhance precision medicine approaches for cardiometabolic and infectious diseases.

## Competing Interests

 The authors declare that there are no conflicts of interest.

## Ethical Approval

 This study does not involve experiments with animals or human participants.
